# Cancer Karyotypes: Survival of the Fittest

**DOI:** 10.3389/fonc.2013.00148

**Published:** 2013-06-07

**Authors:** Joshua M. Nicholson, Daniela Cimini

**Affiliations:** ^1^Department of Biological Sciences, Virginia Tech, Blacksburg, VA, USA

**Keywords:** aneuploidy, karyotype, CIN, cancer, selection

## Abstract

Cancer cells are typically characterized by complex karyotypes including both structural and numerical changes, with aneuploidy being a ubiquitous feature. It is becoming increasingly evident that aneuploidy *per se* can cause chromosome mis-segregation, which explains the higher rates of chromosome gain/loss observed in aneuploid cancer cells compared to normal diploid cells, a phenotype termed chromosomal instability (CIN). CIN can be caused by various mechanisms and results in extensive karyotypic heterogeneity within a cancer cell population. However, despite such karyotypic heterogeneity, cancer cells also display predominant karyotypic patterns. In this review we discuss the mechanisms of CIN, with particular emphasis on the role of aneuploidy on CIN. Further, we discuss the potential functional role of karyotypic patterns in cancer.

## Introduction

The karyotype of a human diploid somatic cell consists of 23 pairs of chromosomes, each encoding different genes essential for cellular function (Alberts et al., 2008). The gain of a single chromosome induces changes in the expression levels of hundreds to thousands of genes, including genes on the extra chromosome as well as genes on other chromosomes (Upender et al., 2004; Nicholson and Cimini, 2011). To limit the generation of aneuploidy in somatic cells, chromosome segregation is tightly regulated during mitosis (Musacchio and Salmon, 2007). Accordingly, chromosome mis-segregation in normal diploid cells occurs at rates below 1% (Cimini et al., 1999; Minissi et al., 1999; Catalan et al., 2000). Cancer cells, which are generally aneuploid (Mitelman et al., 2011), display significantly higher rates of chromosome mis-segregation than normal diploid cells, a phenotype termed chromosomal instability (CIN). Most cancer cells also display chromosome structural instability (S-CIN), by which chromosome aberrations such as translocations, deletions, duplications, etc., occur with high frequencies. This type of instability is different from whole-chromosome mis-segregation/instability (W-CIN), by which numerical defects, such as gains and losses of whole-chromosomes occur at high frequencies. For this review we focus on mechanisms of W-CIN, hereafter referred to as CIN. Both the degree of aneuploidy and the rate of chromosome mis-segregation vary dramatically in cancer cells (Lengauer et al., 1998; Mitelman et al., 2011). Indeed, cancer karyotypes range from near-diploid (2N ± few), to near-triploid (3N ± few), to near-tetraploid (4N ± few), and the rates of chromosome mis-segregation in cancer cells, as measured by anaphase lagging chromosomes, range between 10 and 60% (Thompson and Compton, 2008; Ganem et al., 2009; Silkworth et al., 2009). In this review we will discuss the relationship between aneuploidy and CIN, the karyotype patterns observed in cancer cells, and the effects of such karyotypes on populations of cells or organisms.

## The Effects of Aneuploidy on CIN

**Figure 1:**
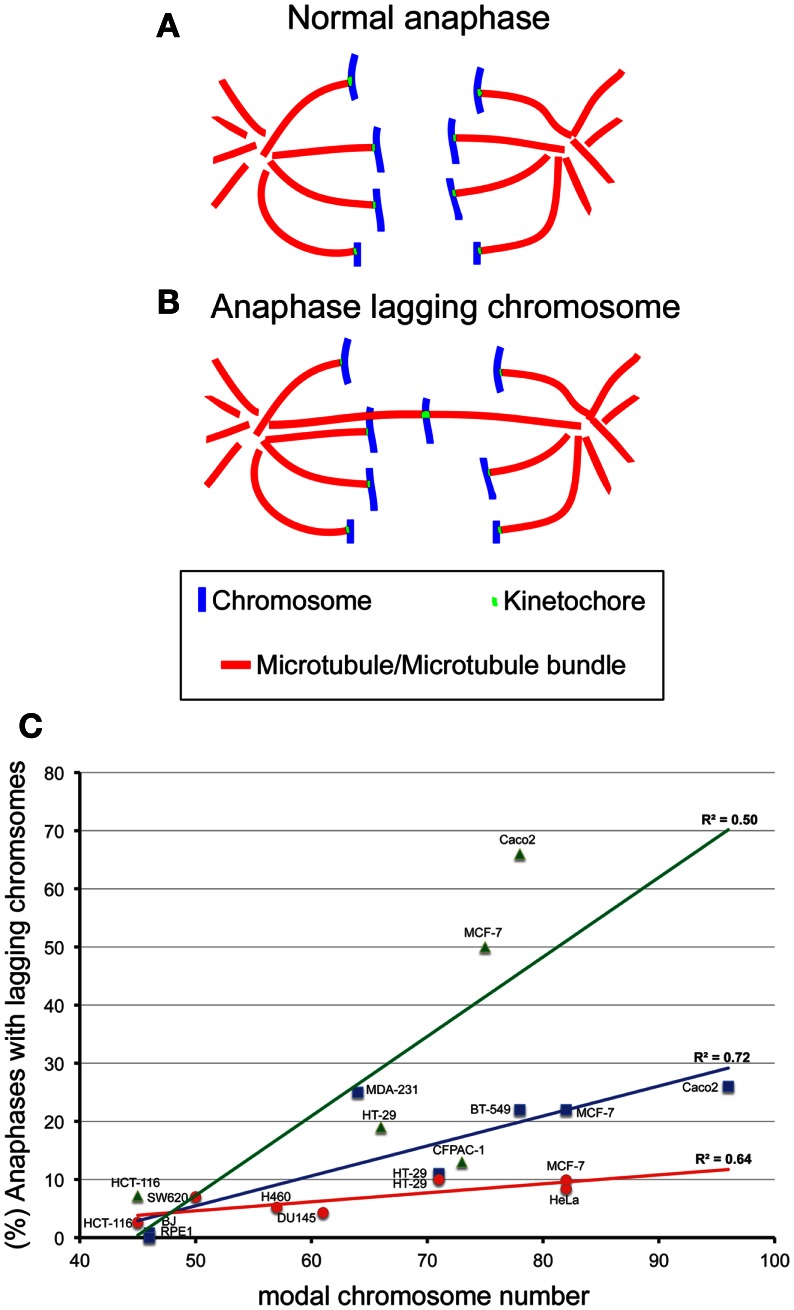
**The degree of aneuploidy directly correlates with CIN, as measured by analysis of anaphase lagging chromosomes**. One way to measure CIN is by determining the rates of anaphase lagging chromosomes in dividing cells. In a normal anaphase, chromosomes are equally segregated to the two poles of the mitotic spindle, as illustrated by the diagram in **(A)**. Some dividing cells display anaphase lagging chromosomes **(B)**, single chromosomes that lag behind at the cell equator as all the other chromosomes move to the spindle poles. Anaphase lagging chromosomes are caused by merotelic kinetochore attachment (Cimini et al., 2001), a kinetochore mis-attachment in which a single kinetochore is bound to microtubules from two spindle poles instead of just one. **(C)** XY plot showing the relation between anaphase lagging chromosomes and modal chromosome number in various cell lines. The graph also shows linear fits and regression values (*R*^2^). The three colors refer to data sets from different labs: red is for data from the Cimini Lab [(Silkworth et al., 2009) and (Silkworth, Nardi, and Cimini, unpublished)]; blue is for data from the Pellman lab (Ganem et al., 2009); green is for data from the Compton lab (Thompson and Compton, 2008). Karyotype information for cell lines from the Cimini Lab and the Pellman Lab was obtained from The American Type Cell Culture website (ATCC). Karyotype information for cell lines from the Compton Lab is that reported in (Thompson and Compton, 2008). Although there is a general trend in which higher chromosome modal number correlates with higher rates of anaphase lagging chromosomes, there is a certain degree of variability between different labs. Correlation analysis showed significant correlation between aneuploidy and CIN for the cell lines in blue (Pearson *R* = 0.85, *P* < 0.05) and those in red (Pearson *R* = 0.80, *P* < 0.05), but no significant correlation for the data shown in green (*R* = 0.71, *P* > 0.05).

Most cancer cells are aneuploid and display a CIN phenotype. CIN can be caused by numerous mechanisms [reviewed in (Nicholson and Cimini, 2011)], including transient spindle geometry defects (Ganem et al., 2009; Silkworth et al., 2009; Silkworth and Cimini, 2012), impaired microtubule dynamics (Bakhoum et al., 2009a,b), and, rarely, a dysfunctional mitotic checkpoint (Cahill et al., 1998; Sato et al., 2000; Haruki et al., 2001), although the mitotic checkpoint is functional in most cancer cells (Tighe et al., 2001). Additionally, abnormal centrosome replication (Lingle et al., 2005) and DNA replication stress (Burrell et al., 2013; Janssen and Medema, 2013) have been proposed as mechanisms of CIN. Abnormal centrosome replication is likely to induce CIN by causing transient spindle geometry defects (Silkworth and Cimini, 2012). However, in the study by Burrell et al. replication stress did not seem related to whole-chromosome mis-segregation. Another mechanism emerging as a cause of CIN in cancer cells is aneuploidy itself (Duesberg et al., 1998; Thompson and Compton, 2010; Sheltzer et al., 2011; Nicholson et al., 2012; Zhu et al., 2012), although there has been disagreement on whether this is really the case, with a number of reports concluding that CIN is an aneuploidy-independent trait (Storchova and Kuffer, 2008; Zasadil et al., 2013). We believe that such disagreements primarily arise from two main issues: (i) there is confusion on how CIN is defined; (ii) different studies measure CIN in different ways. CIN has been loosely defined as an elevated rate of chromosome mis-segregation (Lengauer et al., 1997), yet how elevated and compared to what is often unclear (Geigl et al., 2008). Geigl et al. (2008) suggest that CIN can be defined as a significant increase in the rate of chromosome mis-segregation compared to an appropriate control cell population. Further, appropriate statistical tests must be employed (Geigl et al., 2008). Given this definition, many reports identifying stable aneuploidies can be reinterpreted. Studies that use data available in the Mitelman database of cancer karyotypes (Storchova and Kuffer, 2008; Mitelman et al., 2011; Zasadil et al., 2013) often rely on karyotypic analysis of small numbers (5–20) of cells per cancer, thus masking small rates of CIN that may be present (Adeyinka et al., 1998; Bridge et al., 2004). Other studies lack appropriate statistical analysis (Lengauer et al., 1997). Finally, stable is often used in relative terms. For instance, Roschke et al. (2002) identify stable aneuploid cancer cells in the presence of high rates of chromosome mis-segregation. These cells are considered stable because modal chromosome numbers do not deviate over time, despite deviations per chromosome of up to 20% at a given time (Roschke et al., 2002). The discrepancy between studies concluding that aneuploidy can cause CIN and those concluding that it does not may also stem from the method by which CIN is evaluated/measured in different studies. Many studies measure CIN by one of two methods: (i) performing karyotypic analysis (sometimes simply by chromosome count) at some point in time and measuring what fraction of the cell population possesses a chromosome number that deviates from the mode; (ii) performing FISH staining on interphase nuclei with chromosome-specific probes for two to three chromosomes and again evaluating what fraction of the cell population possesses a number of copies for those chromosomes that deviates from the mode. Neither of these methods really measures chromosome mis-segregation directly and both of them are very likely to underestimate the rates of chromosome mis-segregation occurring at each round of cell division. Because the gain or loss of a single chromosome represents a dramatic genetic change, whether a mis-segregation event can become evident as CIN using one of the methods described above will depend on a number of selective factors, including the specific chromosome that is lost or gained, the specific cell type studied, and the context (e.g., current karyotype, presence/absence of certain environmental conditions, etc.) in which the loss/gain occurs. In other words, cells that mis-segregate chromosomes may or may not survive, and therefore analysis of the karyotype in metaphase spreads or chromosome number in interphase nuclei may reveal a stable karyotype even in the presence of CIN. A more accurate way to measure CIN is by analyzing chromosome segregation in mitotic cells. Many labs have used this approach in recent years and found that CIN cells display higher rates of anaphase lagging chromosomes (chromosomes that lag behind at the cell equator while all other chromosomes segregate to the spindle poles, Figures 1A,B) compared to non-CIN cells (Thompson and Compton, 2008; Bakhoum et al., 2009a; Ganem et al., 2009; Silkworth et al., 2009). Anaphase lagging chromosomes, even when segregated to the correct daughter cell, still represent a mis-segregation event as they typically form micronuclei in the daughter cell (Cimini et al., 2002). Micronuclei have been shown to lead to both numerical and structural defects, including more anaphase lagging chromosomes (Crasta et al., 2012; He et al., 2012). Whereas the analysis of anaphase lagging chromosomes may be a better way to measure CIN, it may still be insufficient to determine the real rates of chromosome mis-segregation, as cases in which two sister chromatids segregate to the same spindle pole would go undetected. A good alternative approach to measure CIN would require the combination of more than one of the methods outlined above, such as, for instance, anaphase lagging chromosomes and interphase FISH or anaphase lagging chromosomes and karyotypic analysis. Alternatively, the analysis of anaphase lagging chromosomes (for all chromosomes) could be combined with analysis of chromosome segregation by FISH with chromosome-specific probes on anaphase cells and/or on binucleate cells in a cytokinesis-block assay (Cimini et al., 1999) and/or in the interphase ensuing cell division (Thompson and Compton, 2008).

Despite the significant difficulties of defining and measuring CIN, many investigators have shown that aneuploidy *per se* can cause CIN. Evidence supporting aneuploidy as a cause of chromosome mis-segregation was first suggested by correlations between the degree of aneuploidy and the degree of CIN in transformed Chinese hamster embryo cells and in colorectal cancer cell lines (Duesberg et al., 1998). Extending this analysis, we have plotted the rate of anaphase lagging chromosomes versus modal chromosome number and found a significant correlation (Figure 1C), indicating that a higher degree of aneuploidy (i.e., modal chromosome number significantly above 46) correlates with higher frequencies of chromosome mis-segregation (i.e., CIN). This, in turn, suggests that aneuploidy can induce chromosome mis-segregation due to an imbalance in the gene dosage (which may include many mitotic genes), such that a higher degree of aneuploidy will result in a more severe imbalance. More controlled analyses of the relationship between aneuploidy and CIN have revealed this relationship to be causal. For instance, studies in haploid yeast strains carrying specific disomies (aneuploidies) showed that aneuploidy induced high rates of chromosome mis-segregation (Sheltzer et al., 2011; Zhu et al., 2012), although not in all disomies tested (70%, 9/13) (Sheltzer et al., 2011), and to varying degrees depending on the specific disomy (Zhu et al., 2012). Moreover, we have recently found that colorectal cancer cells carrying an extra copy of a chromosome (trisomy) also display higher rates of chromosome mis-segregation (anaphase lagging chromosomes and karyotypic heterogeneity) compared to diploid controls (Nicholson et al., 2012). Similar to yeast, we found the effects to vary depending on the specific trisomy (Nicholson et al., 2012). Finally, some studies have reported elevated rates of aneuploidy in somatic cells of individuals affected by congenital trisomies (Reish et al., 2006, 2011). Together, these findings indicate that aneuploidy causes chromosome mis-segregation in the majority of cases. However, not all aneuploidies are capable of doing so, and those that do, do not all do so to the same extent. Given that aneuploidy likely induces chromosome mis-segregation due to the genetic imbalance it generates, it is possible that the differences between different aneuploidies may simply depend on the gene content (both number and types of genes) carried by the aneuploid chromosome. Indeed, studies in disomic yeast show aneuploidy can induce an imbalance in the mitotic checkpoint genes *MAD1* and *MAD2* and in turn increase the rate of chromosome mis-segregation (Zhu et al., 2012). Cancer cells frequently over- or under-express genes involved in mitotic checkpoint and progression, which would be expected to result in an increase in chromosome mis-segregation (Anand et al., 2003; Babu et al., 2003; Yuan et al., 2006; Mondal et al., 2007; Sotillo et al., 2007, 2010; Diaz-Rodriguez et al., 2008; Logarinho et al., 2008; Baker et al., 2009; Ryan et al., 2012). Notably, the genes encoding proteins involved in mitotic checkpoint and progression are typically not mutated, but only mis-expressed in cancer (Cahill et al., 1999; Imai et al., 1999; Yamaguchi et al., 1999; Sato et al., 2000; Haruki et al., 2001). Another mechanism that may explain aneuploidy-induced CIN is a delay in timing of chromosome replication and/or condensation (DRT and DCT, respectively). Pre-mitotic defects such as DRT and DCT have been shown to cause CIN (Smith et al., 2001; Chang et al., 2007; Grinberg-Rashi et al., 2010), and aneuploidy has been shown to induce DRT and DCT (Amiel et al., 1998, 1999; Kost-Alimova et al., 2004) in a chromosome-specific manner. This may depend on the presence of specific loci found on autosomes that control their own stability (Stoffregen et al., 2011; Thayer, 2012). Disrupting these loci leads to a dramatic increase in micro-nucleated cells (Donley et al., 2013), a common outcome of anaphase lagging chromosomes (Cimini et al., 2002) and a common defect of cancer cells (Bhatia and Kumar, 2012). Nonetheless, more work will undoubtedly need to be performed to fully understand how aneuploidy induces CIN.

## The Cancer Karyotype

As described above, most cancer cells display rates of anaphase lagging chromosomes ranging between 10 and 60% (Thompson and Compton, 2008; Ganem et al., 2009; Silkworth et al., 2009). Considering that in 1 cm^3^ of tumor tissue there are approximately 10^9^ cells, chromosome mis-segregation rates of 10–60% could theoretically produce 100,000,000–600,000,000 cells with different karyotypes although it is not known whether these mis-segregation rates identified in cultured cells represent the actual chromosome mis-segregation rates occurring within the tumor. Moreover, although karyotypic analysis has revealed extensive intratumor heterogeneity (Heppner, 1984; Gerlinger et al., 2012), cancer karyotypes are not totally random (Winge, 1930; Levan, 1956; Makino, 1956; Hauschka and Levan, 1958; Roschke et al., 2002; Nicholson and Duesberg, 2009). Indeed, karyotypic analysis of thousands of cancers has revealed the existence of karyotypic patterns, with aneuploidies that are recurrently found in several different cancer types (Table 1), and others that are specific to individual tumors and tissues/organs of origin (Table 1) (Gebhart and Liehr, 2000; Beroukhim et al., 2010; Mitelman et al., 2011; Ozery-Flato et al., 2011; Cai et al., 2012). For instance, extra copies of 1q, 3q, 8q, 7, and 20 are found in numerous different cancers at least 25% of the time (Table 1). And in general, small, gene poor chromosomes are lost across all cancers (Duijf et al., 2012). On the other hand, gain of chromosome 13 is frequently seen in colorectal cancer, but is rarely observed in other cancer types (Bomme et al., 1994; Bardi et al., 1995) (Table 1). Similarly, gain of chromosome 21 is frequent in acute lymphoblastic leukemia, but not in other cancers (Table 1). Karyotype alterations are apparent not only spatially but also temporally, with certain chromosomes being gained or lost earlier than others during cancer progression (Bardi et al., 1995; Fabarius et al., 2002; Ly et al., 2011; Tabach et al., 2011; Ried et al., 2012). What are the factors that generate karyotypic patterns in cancer? Some studies have reported that aneuploidy and CIN can result in loss of heterozygosity for p53 (Matsumura et al., 1992; Blount et al., 1994; Baker et al., 2009) or Rb (Cavenee et al., 1983), supporting the idea that karyotypic changes lead to gain of oncogenes and loss of tumor suppressor genes. Whereas this may be true in some cases, findings from other studies argue against this conclusion. For example, in glioma about half of significant copy number changes are not associated with oncogenes or tumor suppressor genes (Beroukhim et al., 2007). Moreover, if the significance of aneuploidy was exclusively related to its role in promoting the gain of oncogenes or loss of tumor suppressor genes, then one could not explain the individuality of cancer karyotypes per site of origin, given that oncogene and tumor suppressor loci are the same in all human somatic cells. Instead, the fact that the tissue/site of origin is important in determining karyotypic patterns in cancer cells indicates that selection of specific karyotypes must also depend on the specific biology and physiology of cells from different tissues/organs. Indeed, microarray analysis of cells from different tissues shows tissue-specific gene expression patterns in normal diploid cells (Hsiao et al., 2001; Liu et al., 2008). These fixed expression patterns allow cells with the same karyotypes to define different organs and tissues. Such fixed expression patterns will also make it so that the same aneuploidy in different tissues has different effects. Indeed, identical trisomies in different cell types result in different expression patterns for genes both on and off the aneusomic chromosome (Upender et al., 2004). For instance, trisomy 3 in colorectal cancer cells causes significant changes in expression of genes located on chromosomes 1p, 3, 10p, and Y while trisomy 3 in immortalized mammary epithelial cells causes significant changes in expression of genes located on chromosomes 2p, 3, 6q, and 18q (Upender et al., 2004). In further support of the idea that the effect of aneuploidy is cell type-specific is the observation that individuals with trisomy 21 (Down’s syndrome) show an increase in hematological cancers, but decreased incidence of solid tumors compared to diploid individuals (Rabin and Whitlock, 2009). Interestingly, gain of chromosome 21 is the most common karyotype alteration in acute lymphoblastic leukemia (Table 1), but is infrequent in glioblastoma, breast, and colorectal cancer. The enhanced tumorigenic potential conferred by the same aneuploidy in certain tissues but not in others underscores the importance of both the specific karyotypes and the context in which such karyotypes are found.

**Table 1:** **Recurrent aneuploidies in cancers from different sites**.

Cancer type	Recurrent aneuploidies		Cases
	
	Gains	Losses
Acute lymphoblastic leukemia	21	–	533
Breast cancer	**1q**, **8q**, 17q, **20**	**8p**, 16q, **17p**	2,108
Cervical cancer	**3q**	NA	526
Colorectal cancer	**7**, **8q**, 13, **20**	**8p**, **17p**, 18	989
Esophageal cancer	**1q**, **3q**, 5p, **7**, **8q**, 12p, **20**	**3p**, 4, 5q, **8p**, 13, 18q, 19	402
Gastric cancer	**8q**, **20**	–	777
Glioma	**7**	1p, 19q	591
Head and neck cancer	**3q**, **8q**, 11q	**3p**, **8p**	714
Hepatic cancer	**1q**, **8q**	4q, **8p**, 13, 16q, **17p**	903
Medulloblastoma	**7**, 17q	**17p**	1,153
Pancreatic cancer	**1q**, **3q**, 5p, 7p, **8q**, 20q	**3p**, 4q, 6, **8p**, 9p, **17p**, 18, 22	327

Cancers display common recurrent aneuploidies (indicated in bold), as well as recurrent aneuploidies that are tumor – and tissue/organ of origin-specific. Data are adapted from the arrayMap website [www.arraymap.com; (Cai et al., 2012)] and represent gains or losses that occur in at least 25% of cases analyzed.

## The Adaptive Potential of Aneuploidy

The large-scale genomic changes caused by aneuploidy, which alters the expression of hundreds to thousands of genes (Thayer, 1996; Pollack et al., 2002; Upender et al., 2004; Gao et al., 2007), can limit the growth of aneuploid cells under standard environmental conditions (Torres et al., 2007). Under certain circumstances, however, these same changes can confer enhanced fitness (Pavelka et al., 2010; Tang et al., 2011). For example, in *Candida albicans*, formation of isochromosome 5L causes azole resistance via upregulation of the genes ERG11 and TAC1 (Selmecki et al., 2008). Similarly, in aneuploid *Saccharomyces cerevisiae*, the gain of chromosome XIII confers resistance against the DNA damaging agent 4-NQO (Pavelka et al., 2010) due to the overexpression of ATR1, a gene on chromosome XIII whose overexpression is sufficient to confer resistance to 4-NQO (Mack et al., 1988; Pavelka et al., 2010). The adaptive potential of aneuploidy in yeast is well demonstrated for many other exogenous stresses (Selmecki et al., 2006, 2009; Pavelka et al., 2010; Tang et al., 2011; Rancati and Pavelka, 2013). Aneuploidy has also been proposed as a mechanism that can counteract the accumulation of deleterious mutations, a process termed Muller’s Ratchet (Muller, 1964; Bignold, 2007a,b; Torres et al., 2008; Vincent, 2011; Duesberg and McCormack, 2013). An elegant study by Rancati et al. (2008) supports this idea showing that aneuploidy can rescue deleterious mutations in a conserved cytokinesis motor. Similarly, the gain of chromosome VI in yeast is lethal due to the overexpression of the highly dosage-sensitive gene encoding β-tubulin (TUB2) (Katz et al., 1990; Torres et al., 2007; Anders et al., 2009); however, gain of chromosome XIII, which encodes α-tubulin, restores the balance and the double disome VI/XIII is viable (Anders et al., 2009). The findings in yeast extend to many other situations, including naturally occurring chromosomal imbalances, and aneuploidy in cancerous and non-cancerous mammalian cells. For instance, immortalized colon epithelial cells with trisomy 7 out-compete immortalized diploid colon cells in serum-free media (Ly et al., 2011). Likewise, tyrosinemia-induced stress in mouse liver can be overcome by the emergence of aneuploid hepatocytes lacking chromosome 16 (Duncan et al., 2012a). Chromosome 16 carries the homogentisic acid dioxygenase (HGD) gene and loss of HGD in a heterozygous background causes resistance to tyrosinemia-induced hepatic injury (Duncan et al., 2012a). Although the exact mechanisms have been hard to elucidate due to the complexity of cancer karyotypes, the karyotype-phenotype relationship has also been proposed as a mechanism for adaptation in cancer (Duesberg et al., 2007). Indeed, cancer cells that display high rates of CIN, and consequent karyotypic heterogeneity, display intrinsic drug resistance to a wide range of kinase inhibitors (Lee et al., 2011) and other drugs (Li et al., 2005). There have been attempts to selectively inhibit proliferation of aneuploid cells with specific drugs (Tang et al., 2011), however such drugs have not proven effective in other aneuploid cells, even at the highest tolerated dose (Li et al., 2012). In summary, the effects of aneuploidy on the adaptive potential of cancer cells are twofold; (1) specific aneuploidies can confer resistance due to specific changes in gene expression and (2) aneuploidy causes chromosome mis-segregation, thus leading to new karyotypes, some of which will confer selective advantage under specific conditions and in specific contexts.

## The Unique Case of B-Chromosomes: A Naturally Occurring Chromosomal Imbalance

Chromosomal imbalance is generally thought to be associated with disease, which is the case for trisomies causing congenital syndromes, such as Down (trisomy 21), Patau (trisomy 13), and Edwards (trisomy 18) syndrome, or for the high levels of aneuploidy associated with cancer. However, high rates of aneuploidy have also been observed in healthy human tissues, such as liver (Duncan et al., 2012b) and brain (Rehen et al., 2005), in which aneuploidy has been argued to confer adaptive potential (Kingsbury et al., 2005; Duncan et al., 2012a; Bushman and Chun, 2013). Moreover, there exist examples of chromosomal imbalance that simply reflect species-specific evolutionary adaptations. A common example of this is the evolution of sex-specific chromosomes with different genetic content, or the case of species with X0 sex determination system, or the even more extreme case of certain insect species in which a complete set of chromosomes is eliminated (haplodiploidy) in males [for a review on sex determination see (Sumner, 2003)]. Finally, a very unique case is represented by B-chromosomes, extra chromosomes found in the karyotype of wild populations of many animal, fungi, and plant species (Camacho et al., 2000). B-chromosomes are unique supernumerary chromosomes ranging in size and structure from a small fragment to the largest chromosome in the karyotype (Jones and Diez, 2004; Gregory, 2005). While unique, B-chromosomes share some homology with the A-chromosomes (chromosomes of the standard, normal karyotype) and are consequently thought to have derived from them (Jones and Rees, 1982; Martis et al., 2012). The derivation of new chromosomes from the normal chromosome complement is similar to the evolution of marker chromosomes in cancer. Moreover, when present, B-chromosomes can vary in number in different individuals of the same species, thus representing a clear example of chromosomal imbalance similar to aneuploidy. Given the deleterious effect that extra chromosomes can have in many different cell-types and organisms, it is surprising that so many species can maintain these extra chromosomes in their genome. As with aneuploidy, however, this may be possible if B-chromosomes were maintained through a balance between negative effect and adaptive potential. B-chromosomes are largely heterochromatic but they can affect the expression levels of genes on the A-chromosomes (Kirk and Jones, 1970; Ayonoadu and Rees, 1971), and accordingly, induce phenotypic changes such as different sex traits in cichlids (Yoshida et al., 2011) or leaf color in corn (Staub, 1987). The mechanism responsible for maintaining B-chromosomes in the karyotype is still debated (Camacho et al., 2000), but it is clear that, at least in some cases, B-chromosomes can act heterotically (i.e., enhance fitness) and confer an adaptive advantage to the individual carrying them compared to individuals whose karyotype lacks B-chromosomes, much like aneuploidy in yeast grown under stressful conditions. For instance, *Avena sativa* with B-chromosomes shows resistance to rust (Dherawattana and Sadanaga, 1973) and the fungus *Nectria haematococca* with B-chromosomes is resistant to antibiotics (Miao et al., 1991a,b). Similarly, the plant *Allium schoenoprasum* with B-chromosomes displays higher survival rates in natural environments than *A. schoenoprasum* without B-chromosomes (Holmes and Bougourd, 1989), due to the ability of B-chromosomes to enhance the germination rate in drought conditions (Plowman and Bougourd, 1994). Alternatively, B-chromosomes have been thought to be maintained via a “parasitic-selfish model.” In this model, B-chromosomes are detrimental to the carriers (Camacho et al., 2000). In reality, these two models can be unified in a framework in which B-chromosomes, like aneuploidy for a cell, are generally detrimental to the individual, but can confer an adaptive advantage in certain environmental conditions. In support of this model is the observation that in the British grasshopper *Myrmeleotettix maculatus* specific B-chromosomes occur in warm, dry environments, and are scarce or absent in humid, cooler localities (Robinson and Hewitt, 1976).

## Conclusion

That cancer cells are typically aneuploid is currently a widely acknowledged fact. However, there is still confusion on whether most cancer cells also display CIN (i.e., increased rates of chromosome mis-segregation compared to normal cells) or whether this is a less common phenotype. Here we described how the way CIN is typically evaluated is likely to underestimate the actual rates of chromosome mis-segregation in cancer cells. Moreover, emerging evidence indicates that aneuploidy itself promotes CIN. Thus, we argue that CIN, like aneuploidy, is a common feature of cancer cells. One point of confusion about CIN is the fact that cancer karyotypes are not totally random, but rather karyotypic patterns can be identified in various cancers, thus raising the question of how can a cell population mis-segregate chromosomes at high rates and yet display relatively stable karyotypes. We believe that the answer to this question is that karyotype patterns evolve due to the selective pressure within the cancer-specific microenvironment. This will lead to selection of aneuploidies that are common to various cancer types (e.g., chromosomes carrying genes important for cell survival and proliferation in all cell-types) as well as aneuploidies that are specific to individual cancers (e.g., chromosomes carrying genes that are important for cell survival and/or proliferation within a certain tissue/organ). The selection of cancer karyotypes due to the effects they confer (i.e., enhanced fitness in specific environmental conditions/contexts) appear to be the same in naturally occurring chromosomal imbalances (e.g., B-chromosomes), thus indicating that changes in chromosome number represent a natural mechanism of adaptation and evolution or simply stated: survival of the fittest karyotype leads to evolution of cancer, populations, and species.

## Conflict of Interest Statement

The authors declare that the research was conducted in the absence of any commercial or financial relationships that could be construed as a potential conflict of interest.
